# Mistrust and negative self‐esteem: Two paths from attachment styles to paranoia

**DOI:** 10.1111/papt.12314

**Published:** 2020-12-13

**Authors:** Anton P. Martinez, Maximilian Agostini, Azzam Al‐Suhibani, Richard P. Bentall

**Affiliations:** ^1^ Department of Psychology University of Sheffield UK; ^2^ Department of Social and Organizational Psychology University of Groningen The Netherlands; ^3^ Department of Psychology University of Liverpool UK

**Keywords:** attachment, mistrust, paranoia, response bias, self‐esteem, signal detection

## Abstract

**Objectives:**

Paranoia is known to be associated with insecure attachment, with negative self‐esteem as a mediator, but this pathway is insufficient to explain the paranoid individual’s beliefs about malevolent others. Mistrust is a likely additional factor as it is a core feature of paranoid thinking also associated with insecure attachment styles. In this study, we tested whether mistrust – operationalized as judgements about the trustworthiness of unfamiliar faces – constitutes a second pathway from insecure attachment to paranoia.

**Design:**

The design of the study was cross‐sectional.

**Methods:**

A nationally representative British sample of 1,508 participants aged 18–86, 50.8% female, recruited through the survey company Qualtrics, completed measurements of attachment style, negative self‐esteem, and paranoid beliefs. Usable data were obtained from 1,121 participants. Participants were asked to make trustworthiness judgements about computer‐generated faces, and their outcomes were analysed by conducting signal detection analysis, which provided measures of bias (the tendency to assume untrustworthiness in conditions of uncertainty) and sensitivity (accuracy in distinguish between trustworthy and untrustworthy faces).

**Results:**

Results using structural equation modelling revealed a good model fit (*RMSEA* = .071, 95% CI: 0.067–0.075, *SRMR* = .045, CFI = .93, TLI = .92). We observed indirect effects through bias towards mistrust both for the relationship between attachment anxiety and avoidance (β = .003, 95% CI*:* 0.001–0.005,*p* < .001) and attachment anxiety and paranoia (β = .003, 95% CI 0.002–0.006, *p* < .001). We observed an indirect effect through negative self‐esteem only for the relationship between attachment anxiety and paranoia (β = .064, 95% CI*:* 0.053–0.077, *p* < .001). Trust judgements and negative self‐esteem were not associated with each other.

**Conclusions:**

We find that a bias towards mistrust is associated with greater paranoia. We also find indirect effects through bias towards mistrust between attachment styles and paranoia. Finally, we reaffirm the strong indirect effect through negative self‐esteem between attachment anxiety and paranoia. Limitations of the study are discussed.

**Practitioner points:**

- When working with individuals suffering from paranoia, clinicians should consider not only explicit, deliberative cognitive processes of the kind addressed in cognitive behaviour therapy (e.g. cognitive restructuring) but also the way in which their patients make perceptual judgements (e.g., their immediate reactions on encountering new people) and consider interventions targeted at these judgements, for example, bias modification training.
- Assessment and clinical interventions for people should consider the role of trust judgements and the way in which they combine with low self‐esteem to provoke paranoid beliefs.
- Psychological interventions targeting paranoid beliefs should focus on both attachment anxiety and attachment avoidance.

## Background

Paranoid delusions are the most common symptom of psychosis (Bentall, Corcoran, Howard, Blackwood, & Kinderman, 2001) and can be defined as unfounded beliefs characterized by a high degree of conviction, pre‐occupation, and distress in which the core theme includes intentional harm to the person who is holding the belief (American Psychiatric Association, 2013). However, less severe forms of paranoid beliefs are also experienced by at least 10–15% of the general population (Freeman, 2007) suggesting that clinical paranoia lies on a continuum with subtle subclinical forms (Bebbington et al., 2013; Elahi, Perez Algorta, Varese, McIntyre, & Bentall, 2017). These lesser forms of paranoid beliefs, although held with less conviction in comparison to clinical paranoid delusions, are still associated with distress, social isolation, and feelings of powerlessness (Freeman et al., 2005). By understanding the precursors of paranoid beliefs in the general population, we may therefore gain an understanding of mechanisms that may be responsible for more severe forms in clinical groups.

Consistent with the hypothesis that attachment processes may play a role in the development of paranoid beliefs (Bentall & Fernyhough, 2008), many studies have reported a strong association between insecure attachment styles and paranoia in both clinical and non‐clinical samples (Carr, Hardy, & Fornells‐Ambrojo, 2018; Gumley, Taylor, Schwannauer, & MacBeth, 2014; Pickering, Simpson, & Bentall, 2008; Ringer, Buchanan, Olesek, & Lysaker, 2014; Sitko, Bentall, Shevlin, O’Sullivan, & Sellwood, 2014; Wickham, Sitko, & Bentall, 2015). These styles can be conceptualized as internalized representations of relationships with primary caregivers that take the form of working models of the self and others which in turn guide interpersonal behaviour (Bowlby, 1982, 2015; Shaver & Mikulincer, 2005). In adulthood, attachment styles can be secure or insecure, the former reflecting confidence in the availability of attachment figures and the latter reflecting the contrary. Two underlying dimensions of attachment security/insecurity in adults are anxiety and avoidance (Mikulincer, 1995). Attachment avoidance is associated with insecurity about other’s intentions, preference for emotional distance, and a negative view of others; attachment anxiety reflects negative self‐image, fear of rejection, and excessive need of approval (Berry, Wearden, & Barrowclough, 2007; Mikulincer, 1995).

Several cross‐sectional studies have reported that the association between insecure attachment styles – particularly attachment anxiety – and paranoia is mediated by negative self‐esteem (Pickering et al., 2008; Ringer et al., 2014; Wickham et al., 2015). In longitudinal (experience sampling) studies, paranoid symptoms are predicted by fluctuations in self‐esteem (Thewissen, Bentall, Lecomte, van Os, & Myin‐Germeys, 2008) and attachment‐related cognitions (Sitko, Varese, Sellwood, Hammond, & Bentall, 2016). Self‐esteem involves the evaluation of attributes of the self (Hahn & Gawronski, 2015) which are influenced by internal working models about the self and others (i.e., attachment styles; Sitko et al., 2016). Hence, negative internal working models lead to negative evaluations about the self which in turn lead to feelings of vulnerability and the anticipation of social threats (Bentall & Fernyhough, 2008). However, based on the observation that the mediating role of self‐esteem is also present in the association between attachment anxiety and depression symptoms (Lee & Hankin, 2009; Roberts, Gotlib, & Kassel, 1996), the relationship between insecure attachment and paranoia would seem to require the involvement of additional factors. Mistrust is a likely candidate for this relationship, as it is a subcomponent of the paranoia spectrum often present in subclinical populations (Bebbington et al., 2013; Bell & O’Driscoll, 2018), and insecure attachment styles are associated with reduced interpersonal trust (Fett et al., 2016; Mikulincer, 1995, 1998).

### The role of trustworthiness judgements in paranoid beliefs

During everyday life, most people meet numerous persons and judgements of trustworthiness have to be made rapidly without effortful deliberation (Sutcliffe, Dunbar, Binder, & Arrow, 2012). Trustworthiness judgements are a dominant mode of appraisal when encountering unfamiliar faces (Oosterhof & Todorov, 2008). Healthy individuals typically make judgements of trust very quickly – within a few hundred milliseconds – and consistently rate some unfamiliar faces as less trustworthy than others (Todorov, Pakrashi, & Oosterhof, 2009). However, studies of trustworthiness judgements in patients with a schizophrenia diagnosis (for which paranoia is a prominent symptom) have shown inconsistent findings. On the one hand, in one study participants diagnosed with schizophrenia judged unfamiliar face images to be more trustworthy than controls (Baas, van't Wout, Aleman, & Kahn, 2008), potentially as a consequence of reduced social cognitive abilities (Green et al., 2008). Conversely, in another study, after being negatively primed with threat‐related images, participants with a schizophrenia diagnosis judged face stimuli as more untrustworthy in comparison with non‐clinical controls (Hooker et al., 2011); however, this difference was not present in neutral priming conditions. Using computer‐generated face stimuli (Todorov, Dotsch, Porter, Oosterhof, & Falvello, 2013), another study found that participants with high paranoia ideation judged unfamiliar faces as less trustworthy than those with low paranoia but that this was true for faces previously calibrated to appear trustworthy, untrustworthy, or neutral, suggesting a general bias towards mistrust (Kirk, Gilmour, Dudley, & Riby, 2013).

### Signal detection theory and trustworthiness

Signal detection theory (SDT; Swets, Dawes, & Monahan, 2000) offers a useful framework for analysing behavioural decision‐making outcomes when judging trustworthiness of faces that have been previously selected to appear trustworthy or untrustworthy. There are four possible outcomes: hits (in this case, identifying a trustworthy face when a trustworthy face is present); false alarms (identifying a trustworthy face when a non‐trustworthy face is present); correct rejections (identifying a non‐trustworthy face when a non‐trustworthy face is present); and misses (identifying a non‐trustworthy face when a trustworthy face is present). These decision outcomes can be operationalized into two components (Stanislaw & Todorov, 1999): (1) *response bias,* which reflects the general tendency to respond ‘Yes’ (e.g., trustworthy) versus ‘No’ (e.g., non‐trustworthy) and (2) *sensitivity,* which mirrors the perceiver’s ability/accuracy to discriminate between a target and a non‐target stimulus (e.g., make correct vs. incorrect decisions; Lynn & Barrett, 2014).

Whilst decreased sensitivity might indicate a deficit in information processing (Frith, 1979), response biases are sensitive to the differential costs and benefits of the different response outcomes (Correll, Park, Judd, & Wittenbrink, 2002; Haselton & Buss, 2000) and are hypothesized to be evolutionary adaptive strategies for minimizing the more costly type of error in uncertain and complex environments – for example perceiving non‐trustworthy faces as trustworthy (Haselton & Nettle, 2006). Therefore, response bias may suggest a tendency towards a liberal criterion for detecting threat or a strict criterion for detecting non‐threat (Haselton & Buss, 2000). Consistent with this account, studies have shown that paranoid participants adopted a liberal response bias for detecting angry faces after viewing anxiety evoking pictures, although not when not anxious (Westermann & Lincoln, 2010). Similarly, in another study participants diagnosed with schizophrenia showed a liberal response criterion when recognizing fear and sad emotions in comparison to controls (Tsoi et al., 2008).

### Aims of the current study

This study has two main aims: First, we will implement signal detection theory to test the association between judgements of mistrust and paranoia in non‐clinical participants; we hypothesize that a response bias towards judging faces as untrustworthy, but not sensitivity to trustworthiness cues, will be associated with paranoia. Second, we aim to test whether there is an indirect effect of attachment on paranoia through mistrust bias; given that self‐esteem involves the evaluation of the self (Nugent & Thomas, 1993), whereas judgements of trustworthiness involve the evaluation of others (Burns & Conchie, 1982, 2015), we hypothesize that this indirect effect will be separate and independent of the already established indirect effect through negative self‐esteem. Moreover, we hypothesize that mistrust will be particularly associated with attachment avoidance since this style involves a negative working model of others (Fett et al., 2016).

## Methods

### Participants

We recruited a nationally representative sample of 1,508 British participants for a multipurpose survey, age range 18–86 (*M = *47.8, *SD = *17.2), 50.7% female, through the survey company Qualtrics. Participants were stratified on the basis of the Office for National Statistics data by age, sex, and household income.^1^ Regarding ethnicity, 89% of the sample was white British/Irish, whereas the remaining 11% consisted of white non‐British/Irish (2.2%), Indian (1.8%), Pakistani (1.3%), Chinese (0.9%), Other‐Asian (0.9%), Afro‐Caribbean (0.7%), African (0.7%), Bangladeshi (0.7%), and other ethnic groups (1.8%).

An additional 344 participants were removed due to incomplete survey responses or completing the survey implausibly quickly (our pre‐defined cutoff criteria based on pilot work and recommended by the survey company was 12 min).

### Procedure

After completing informed consent, participants filled out a number of measures regarding self‐esteem, attachment styles, and paranoid beliefs before engaging in the facial trust detection task.

### Measures

*The revised Paranoia and Deservedness Scale* (PaDS –R) was designed on the basis of psychometric analyses of the original scale (Melo, Corcoran, Shryane, & Bentall, 2009) in a large sample of healthy individuals and patients with psychosis (Elahi et al., 2017). In line with recent findings (Bebbington et al., 2013), the 8‐item scale measures the four elements of paranoia (two items per element): interpersonal sensitivity (e.g., ‘My friends often tell me to relax and stop worrying about being deceived or harmed’), mistrust (e.g., ‘You should only trust yourself’), fear of persecution (e.g., ‘I believe that some people want to hurt me deliberately’), and ideas of reference (e.g., ‘Sometimes I think there are hidden insults in things that other people say or do’). Items were answered on a 5‐point scale ranging from ‘Strongly disagree’ to ‘Strongly agree’. Scale reliability was good (α = .87), and responses were normally distributed.

*The Relationship Questionnaire* (RQ) was used to assess attachment style (Bartholomew & Horowitz, 1991). Participants read four vignettes describing secure, fearful, pre‐occupied, and dismissing prototypical styles and had to choose the one that describes them best. They were then asked to rate each vignette ‘according to how well or poorly each description corresponds to [their] general relationship style’ on 7‐point scales ranging from ‘Disagree strongly’ to ‘Agree strongly’. Scores on the four scales were used to compute higher order measures of attachment anxiety (negative model of self) by subtracting the sum of secure and dismissing scores from the sum of pre‐occupied and fearful scores and attachment avoidance (negative model of other) by subtracting the sum of secure and pre‐occupied scores from the sum of dismissing and fearful scores. Thus, the formula can be summed up as follows: model of self = (secure + dismissing) − (pre‐occupied + fearful); model of other = (secure + pre‐occupied) − (dismissing + fearful), where higher scores indicate the presence of each type of attachment style.

*The short version of the Self‐esteem rating scale* (SERS; Lecomte, Corbière, & Laisné, 2006) is a 20‐item scale, designed to assess self‐esteem independently of mood. It consists of 10 positive statements about the self, for example, ‘I feel good about myself’, and 10 negative statements about the self, for example, ‘I feel that others do things much better than I do’. Participants rated each statement from 1, ‘*never*’, to 7, ‘*always*’. For both positive self‐esteem (α = .94) and negative self‐esteem (α = .94), scale reliability was good. However, because negative self‐esteem rather than positive self‐esteem has been found to be a strong predictor of paranoia in previous studies (Bentall et al., 2008), only negative self‐esteem is considered in this study.

**Figure 1:**
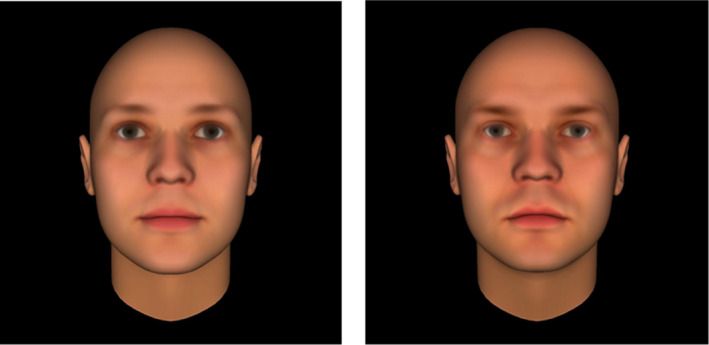
Left image example of a trustworthy computer‐generated face. Right image example of an untrustworthy computer‐generated face (Images obtained from the Princeton Social Perception Lab database).

*Facial trust detection task* was based on the trustworthiness data set (25 identities; Oosterhof & Todorov, 2008). This data set contains computer‐generated faces created using FaceGen 3.1 and obtained from the Princeton Social Perception Lab database.^2^ The database includes identities manipulated on different traits (attractiveness, competence, dominance, extroversion, likeability, threat, and trustworthiness). From this data set, 10 bald Caucasian male computer‐generated faces (5 prior rated as trustworthy and 5 prior rated as untrustworthy; see Figure 1) were randomly selected by using the website www.Random.org. Participants were presented with each face followed by a fixation cross and were asked: ‘How much would you trust this person’. Answers were given on a 7‐point Likert scale (1 = *‘I would not trust this person at all*’ to 7 = ‘*I would trust this person completely’*). Reliabilities were good for the overall scale (α = .94), the trustworthy items (α = .93), and the untrustworthy items (α = .93). In the sample as a whole, the trustworthy faces were rated as more trustworthy (*M* = 20.10, *SD* = 5.58) than the untrustworthy faces (*M* = 16.49, *SD* = 5.62, *t* = 30.56, *p* < .001), showing that the faces were clearly discriminable. However, mean ratings for the two types of faces were correlated, *r* = .66, *p* < .001, suggesting individual differences in trust judgements.

### Signal detection analysis

In order to analyse facial trust detection task outcomes using a signal detection paradigm, participant’s responses were recoded as binary outcomes (a total of 15,080 trials for all participants) by recoding responses from 1 to 3 as a *NO* outcome (5,603 trials for all participants), responses from 5 to 7 as a *YES* outcome (3,524 trials for all participants), and responses of 4 as null outcomes (5,953 trials for all participants), so that positive values reflect bias towards trustworthiness. The amount of null responses resulted in a loss of 387 participants for which we were unable to calculate signal detection variables due to the inability of distinguishing yes and no responses. This meant our final sample size was reduced to 1,121 participants.

Signal detection outcomes were analysed based on Stanislaw and Todorov's (1999) calculations using equation 7 for response bias: *c* = $\frac{\Phi^{‐1}\left(H\right)+\Phi^{‐1}\left(F\right)}{2}$ where $\Phi^{‐1}$ (‘inverse phi’) function converts hit (H) rates (dividing the number of hits by total number of signal trials) and false alarm (F) rates (dividing the number of false alarms by total number of noise trials) into *z* scores. This measure evaluates whether people have a bias towards pressing the ‘trust’ or ‘not trust’ button. Sensitivity calculations were based on Stanislaw and Todorov’s equation 1: *d*′= $\Phi^{‐1}\left(H\right)‐\Phi^{‐1}\left(F\right)$ with adjustments for potential assumption violations^3^ (see Stanislaw & Todorov, 1999 for details). The sensitivity measure evaluates whether people are accurate in identifying whether faces are trustworthy or non‐trustworthy; hence, it is a measure of sensitivity to trustworthiness cues. As we were also interested in specific errors on judgement that trustworthy faces were non‐trustworthy (misses) and that non‐trustworthy faces were trustworthy (false alarms), these were also separately recorded and reported. Although sensitivity and response bias measures are superior measures, false alarms and misses are inherently easier to interpret.

### Statistical analysis

**Figure 2:**
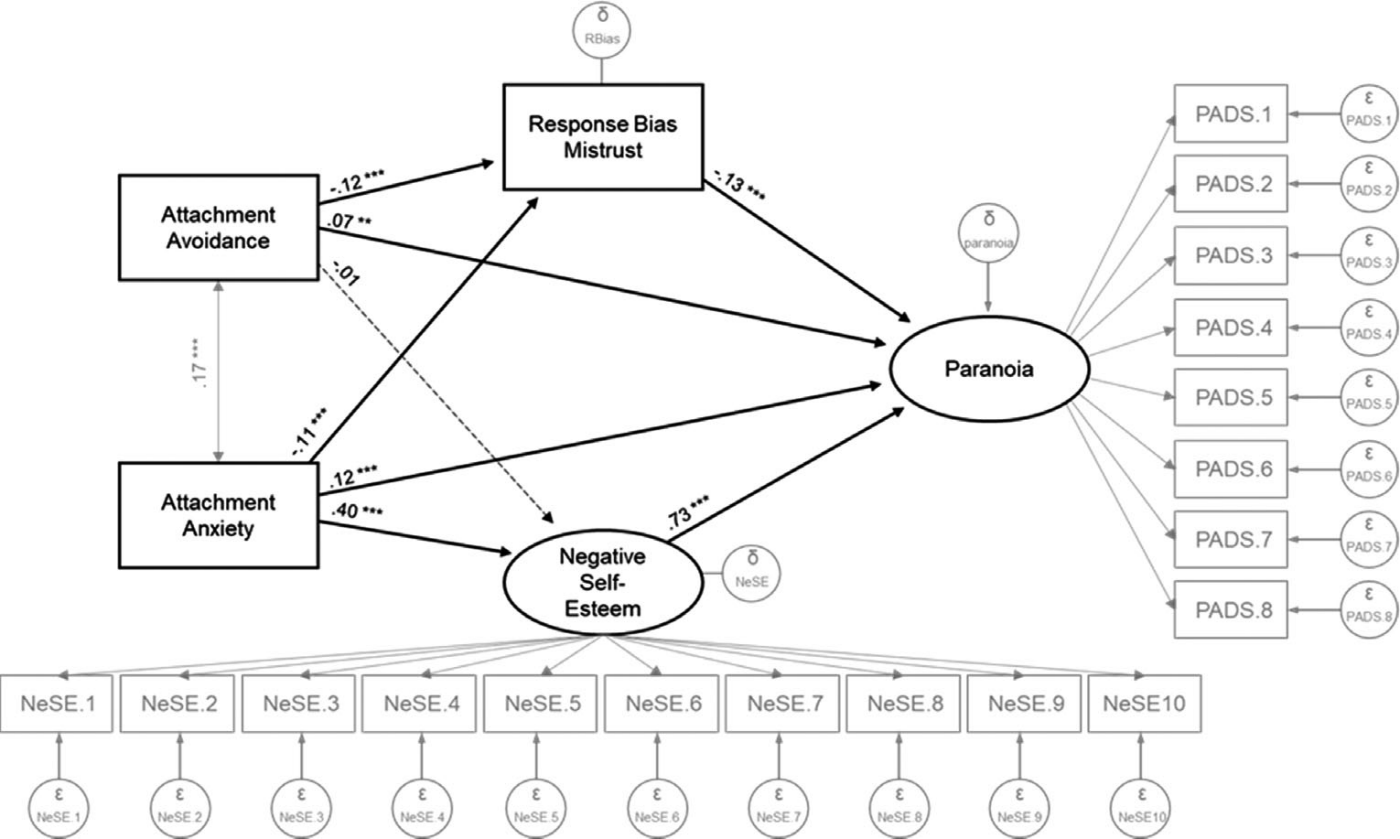
Full mediation model between attachment styles, response bias towards mistrust, negative self‐esteem, and paranoia traits. All estimates are standardized. Level of significance **p < .01, ***p < .001.

Analyses were carried out in R 1.1.463, using CAR package *lm* function for linear regression and *cor* for correlations (Fox & Weisberg, 2019; Hlavac, 2018; R Core Team, 2018; RStudio Team, 2016). Our first aim was to assess whether signal detection variables (sensitivity and bias) correlated with paranoia. To test our second, indirect effects hypothesis, we implemented a structural equation model in AMOS 25.0.0. For this model, attachment avoidance and attachment anxiety were modelled as independent variables, negative self‐esteem, and trust judgement response bias were included as indirect effect/mediator variables, and paranoia was the outcome variable. We modelled negative self‐esteem and paranoia as latent constructs and trust and attachment as observed variables since paranoia and self‐esteem were measured through items tapping into their respective latent constructs. Attachment styles and signal detection variables were measured directly either through vignettes or the face rating task and were therefore modelled as observed variables; see Figure 2. Following suggestions of Kline (2015), we report five goodness of fit indices: the chi‐square test; root mean square error of approximation (RMSEA; MacCallum, Browne, & Sugawara, 1996); standardized root mean squared residual (SRMR, note: inflated with large sample sizes; Hu & Bentler, 1999); the comparative fit index (CFI); and Tucker–Lewis Index (TLI). We also report bootstrap bias‐corrected confidence intervals (based on 5,000 bootstrap samples) to avoid problems of non‐normal data.

## Results

### Association between paranoia and trustworthiness judgements

Correlational analyses showed that there were only trivial associations between self‐esteem and the face judgement measures (*r *= −.09; Table 1). Higher levels of paranoia were positively related to misses (i.e., judging a face as untrustworthy when the target is trustworthy). Higher levels of paranoia were also negatively associated with false alarms (i.e., judging a face stimulus as trustworthy when it is non‐trustworthy). Using signal detection measures, there was a stronger association between paranoia and response bias (*r* = −.20) than between paranoia and sensitivity (*r* = −.10); *z*(1,508) = 3.10, *p* = .002 (Lee & Preacher, 2013) although, contrary to expectation, the latter association was significant.

**Table 1:** Bivariate correlations between main variables with means and standard deviations

Variables	*M*	*SD*	1	2	3	4	5	6	7	8
1. Paranoia	2.78	1.00	–	.41**	.14**	.74**	−.10**	−.20**	−.13**	.21**
2. Attachment anxiety	−0.87	3.50		–	−.17**	.39**	−.03	−.13**	−.10**	.12**
3. Attachment avoidance	0.85	3.57			–	.05	−.04	−.14**	−.11**	.13**
4. Negative self‐esteem	3.35	1.43				–	−.09**	−.09**	−.03	.11**
5. Sensitivity	0.67	0.22					–	.19**	−.38**	−.61**
6. Response bias	−0.31	1.15						–	.83**	−.88**
7. False alarms	0.026	0.33							–	−.47**
8. Misses	0.44	0.40								–

Note

* *p* < .05

** *p* < .01

*** *p* < .001.

These findings were confirmed using regression analyses. Sensitivity and response bias (both centred) predicted a significant proportion of variance in paranoia, *R* ^2^_adj_ = .04, *F*(2, 1,118) = 26.30, *p* < .001. Higher sensitivity, *b* = −.30 95%‐CI [−0.57, −0.04], *t*(1,118) = −2.25, *p* = .02, and a stronger response bias towards trustworthiness, *b* = −.16 95%‐CI [−0.21, −0.11], *t*(1,118) = −6.36, *p* < .001, predicted lower paranoia scores. It is important to note that, because sensitivity and bias are measured on different scales, this finding does not imply a greater effect for sensitivity. When standardized for comparison, we see that the response bias, *b* = −.19, 95%‐CI [−0.25, −0.13], *t*(1,118) = −6.36, *p* < .001, is in fact a stronger predictor of paranoia than sensitivity, *b* = −.07, 95%‐CI [−0.13, −0.009], *t*(1,118) = 2.25, *p* =.02.^4^

### Indirect effects analysis

The model chi‐squared test was statistically significant, χ^2^(183, *N* = 1,121) = 1,209.93, *p* < .001, which was to be expected given the large sample size (Kenny, 2019). The other recommended fit indexes suggested good model fit, with the absolute fit measures *RMSEA* = .071, 95%‐CI 0.067–0.075, and *SRMR* = .045 being smaller than the recommended .08. The *CFI* = .93 and *TLI* = .92 were above the .9 rule of thumb.

We observed significant direct effects for all of our paths other than the path from attachment avoidance to negative self‐esteem, β = −.01, 95% CI −0.07, −0.05, *p* = .77 (Figure 2). Importantly, as in previous work (Wickham et al., 2015), the path from attachment anxiety to negative self‐esteem, *β* = .40, 95% CI 0.35–0.45, *p* <.001, and the path from negative self‐esteem to paranoia, β = .73, 95% CI 0.68–0.77, *p* < .001, were the strongest.

In terms of indirect/mediation effects, we observed a strong indirect effect between attachment anxiety on paranoia through negative self‐esteem, β = .064, 95% CI 0.053–0.077, *p* < .001, and a weaker indirect effect of attachment anxiety on paranoia through a response bias towards mistrust, β = .003, 95% CI 0.001–0.005, *p* < .001. For the indirect effect of attachment avoidance on paranoia, we found no effect through negative self‐esteem, β = −.002, 95% CI −0.011 to 0.008, *p* =. 78, and a weak effect through response bias, β = .003, 95% CI 0.002–0.006, *p* < .001.

To discriminate whether the indirect effect went through the response bias or sensitivity measure, we first added sensitivity as a third mediator. This undermined model fit considerably. We then replaced response bias with sensitivity. None of the direct effects from attachment styles to sensitivity, nor the direct effect from sensitivity to paranoia, nor any of the indirect effects through sensitivity were significant; all *p*s > .15 and β*<* .03 (also see Figure S1). This suggests that the indirect effect from attachment styles on paranoia goes through response bias but not sensitivity.

## Discussion

In this study, our first aim was to assess whether paranoid traits are associated with judgements of untrustworthiness operationalized as signal detection outcomes (bias and sensitivity), hypothesizing an association with bias but not sensitivity. Unexpectedly, we observed that paranoid traits were associated with reduced sensitivity, suggesting a reduced ability to detect subtle facial cues that signal trustworthiness. Nonetheless, in our subsequent regression analyses response bias was the stronger and more stable predictor. This finding suggests a tendency towards a liberal criterion for initiating a threat‐related response (e.g., perceiving a trustworthy face as untrustworthy; Correll et al., 2002; Haselton & Buss, 2000) and is consistent with other evidence that people with high paranoia traits show an increased tendency to anticipate social threat (Bentall et al., 2009). Previous studies have addressed the association between judgements of faces and paranoia in clinical and non‐clinical populations revealing inconsistent findings either by suggesting that patients diagnosed with schizophrenia might have difficulties discriminating facial stimuli (Baas et al., 2008) or proposing that participants with high paranoia traits show a bias towards mistrust (Kirk et al., 2013). However, none of these studies assessed mistrust outcomes from a signal detection perspective. Thus, to the best of our knowledge, this is the first study that analyses judgements of trust outcomes using a signal detection framework.

As our second aim, we wanted to expand our understanding of the psychological mechanisms that mediate between insecure attachment and paranoia (Bentall & Fernyhough, 2008; Pickering et al., 2008; Wickham et al., 2015). For this purpose, we considered bias towards mistrust as a second mediator that is independent of negative self‐esteem. In line with our second hypothesis, we found indirect effects of attachment anxiety on paranoid beliefs through both bias towards mistrust as well as negative self‐esteem. As stated in our third hypothesis, the indirect effect from attachment avoidance on paranoia went only through mistrust. Attachment avoidance reflects negative view of others, whereas attachment anxiety reflects a negative view of oneself (Fett et al., 2016) and, hence, it is perhaps unsurprising that the mediating effect for self‐esteem was only found in the case of anxiety. When we substituted sensitivity for response bias, the model did not hold. Together, these findings suggest that response bias plays a larger role than sensitivity in explaining the association between insecure attachment styles and paranoia and that mistrust is an additional component of paranoia that is independent of self‐esteem.

Several authors suggest that dysfunctional attachment styles, as a result of repeated experiences of victimization, are likely to heighten negative self‐esteem and thus contribute to the feeling of being vulnerable to the actions of powerful others (Bentall & Fernyhough, 2008; Freeman, 2007). Moreover, childhood attachment disruption experiences may limit the availability of secure attachment figures, leading to feelings of mistrust of others (Mikulincer, 1995; Sitko et al., 2016). Consistent with these accounts, empirical research supports an association between disrupted early attachment relationships and paranoia (Bentall, Wickham, Shevlin, & Varese, 2012; Varese et al., 2012) and suggests that insecure attachment helps to explain this association (Sitko et al., 2014). Given that trust judgements dominate initial evaluations of new people, we expected that, when considered from a decision‐making perspective, the feelings of mistrust created by an insecure attachment style would be manifest in a bias towards assuming that novel faces are untrustworthy. Although the design of our study was cross‐sectional, our results point to how this attachment‐based model can be expanded to account for the negative beliefs about the self and the intentions of others that are the key feature of paranoid thinking. Future research would benefit by employing experimental and longitudinal designs to establish causality whilst incorporating measures of childhood adversity, attachment, and mistrust.

### Limitations

We acknowledge a number of limitations of this study. In terms of the facial trust detection ask, our mass online testing allowed us to present only ten faces to participants, which may have limited the precision of our signal detection measures (Essien et al., 2017); in future research, it will be useful to employ more trials. A second limitation was that we only used male Caucasian faces. This decision was made in the light of evidence that there is a bias to classify bald, hairless faces as males (Todorov et al., 2013); thus, it is possible that our findings may not extend to females faces as well as faces from different ethnic backgrounds. Moreover, the use of computer‐generated faces as stimuli might have limited the ecological validity of our study, and it would be useful to replicate our findings with, for example, video‐recorded images of real people. A third limitation is the large number of participants who had to be excluded because they consistently indicated ‘4: neutral’ on the facial trust detection task, which may have reflected failure to engage with it. Our cross‐sectional data allow only limited capability to make causal statements, and our findings should be seen as consistent with an attachment‐based developmental pathway rather than proving the existence of such a pathway. Finally, the effect sizes for the mistrust pathway were smaller than the effect sizes for the self‐esteem pathway. It would be tempting, but in our view premature, to assume that the self‐esteem pathway is more important. Small effect sizes between signal detection operationalizations and self‐report scales are common in research (Mekawi & Bresin, 2015), and these kinds of effects can have large societal implications at the population level (see, Mekawi & Bresin, 2015). Our small effects may be also a reflection of our methods. We would expect larger effects in a controlled laboratory environment and in clinical samples. This being said, our large and highly representative sample allows us to generalize to the UK population and allowed us to conduct high‐powered statistical tests.

### Conclusions

To the best of our knowledge, this is the first study to examine the relationship between mistrust operationalized as signal detection outcomes and paranoid beliefs. Our results revealed that participants with high paranoia traits show a bias towards mistrust when judging unfamiliar faces and that this process was also associated with insecure attachment styles. Moreover, our findings also revealed an indirect mediating effect of negative self‐esteem between attachment anxiety and paranoia but not between attachment avoidance and paranoia. Although future research is needed to replicate these findings and to establish the direction of these associations, these findings should encourage clinicians to consider the role of mistrust in clients who are experiencing paranoia and to develop interventions for these patients that specifically target insecure attachment and trust judgements in combination with already established interventions for self‐esteem.

## Conflicts of interest

All authors declare no conflict of interest.

## Author contributions

Anton Martinez (Conceptualization; Formal analysis; Methodology; Writing – original draft) Maximilian Agostini (Formal analysis; Methodology; Writing – original draft) Azzam Al‐Suhubani (Conceptualization; Funding acquisition; Investigation; Software; Writing – review & editing) Richard Bentall (Conceptualization; Funding acquisition; Investigation; Methodology; Supervision; Writing – original draft; Writing – review & editing).

## Supporting information

**Figure S1.** The only significant indirect effect was attachment anxiety → negative self‐esteem → paranoia, β = .065, 95% CI 0.055–0.077, *p* < .001.

## Data Availability

The data that support the findings of this study are available from the corresponding author upon reasonable request.

## Footnotes

^1^

https://www.ons.gov.uk/peoplepopulationandcommunity/personalandhouseholdfinances/incomeandwealth/bulletins/householddisposableincomeandinequality/financialyearending2016

^2^

http://tlab.princeton.edu/databases/secretdatabaseportal/

^3^
Extreme values (0s and 1s) were adjusted following using the approach of Stanislaw and Todorov (1999; see p. 144), in which rates of 0s are replaced with 0.5/*n*, and rates of 1s are replaced with (*n *− 0.5)/*n*, where *n* is the number of signal or noise trials.

^4^
Standardization does not always allow for direct comparison of effects (King, 1986). However, as the estimate of the response bias when considering its confidence interval falls further away from zero, we can be more confident that in the long run its true estimate may fall more often outside of 0. Overall, the results hint towards response bias being a stronger predictor.

## References

[papt12314-bib-0001] American Psychiatric Association (2013). Diagnostic and statistical manual of mental disorders (5th ed.). Washington, DC: American Psychiatric Pub. 10.1176/appi.books.9780890425596

[papt12314-bib-0002] Baas, D., van't Wout, M., Aleman, A., & Kahn, R. S.(2008). Social judgement in clinically stable patients with schizophrenia and healthy relatives: Behavioural evidence of social brain dysfunction. Psychological Medicine, 38, 747–754. 10.1017/S0033291707001729 17988413

[papt12314-bib-0003] Bartholomew, K., & Horowitz, L. M. (1991). Romantic love conceptualized as an attachment process. Journal of Personality and Social Psychology, 61(2), 226–244. Retrieved from http://www.ncbi.nlm.nih.gov/pubmed/1920064 1920064 10.1037//0022-3514.61.2.226

[papt12314-bib-0004] Bebbington, P. E., McBride, O., Steel, C., Kuipers, E., Radovanovič, M., Brugha, T., … Freeman, D. (2013). The structure of paranoia in the general population. British Journal of Psychiatry, 202, 419–427. 10.1192/bjp.bp.112.119032 23661767

[papt12314-bib-0005] Bell, V., & O’Driscoll, C. (2018). The network structure of paranoia in the general population. Social Psychiatry and Psychiatric Epidemiology, 53, 737–744. 10.1007/s00127-018-1487-0 29427197 PMC6003969

[papt12314-bib-0006] Bentall, R. P., Corcoran, R., Howard, R., Blackwood, N., & Kinderman, P. (2001). Persecutory delusions: A review and theoretical integration. Clinical Psychology Review, 21, 1143–1192. 10.1016/S0272-7358(01)00106-4 11702511

[papt12314-bib-0007] Bentall, R. P., & Fernyhough, C. (2008). Social predictors of psychotic experiences: Specificity and psychological mechanisms. Schizophrenia Bulletin, 34, 1012–1020. 10.1093/schbul/sbn103 18703667 PMC2632492

[papt12314-bib-0008] Bentall, R. P., Rouse, G., Kinderman, P., Blackwood, N., Howard, R., Moore, R., … Corcoran, R. (2008). Paranoid delusions in schizophrenia spectrum disorders and depression: The transdiagnostic role of expectations of negative events and negative self‐esteem. Journal of Nervous and Mental Disease, 196, 375–383. 10.1097/NMD.0b013e31817108db 18477879

[papt12314-bib-0009] Bentall, R. P., Rowse, G., Shryane, N., Kinderman, P., Howard, R., Blackwood, N., … Corcoran, R. (2009). The cognitive and affective structure of paranoid delusions: a transdiagnostic investigation of patients with schizophrenia spectrum disorders and depression. Archives of General Psychiatry, 66(3), 236–247. 10.1001/archgenpsychiatry.2009.1 19255373

[papt12314-bib-0010] Bentall, R. P., Wickham, S., Shevlin, M., & Varese, F. (2012). Do specific early‐life adversities lead to specific symptoms of psychosis? A study from the 2007 the adult psychiatric morbidity survey. Schizophrenia Bulletin, 38, 734–740. 10.1093/schbul/sbs049 22496540 PMC3406525

[papt12314-bib-0011] Berry, K., Wearden, A., & Barrowclough, C. (2007). Adult attachment styles and psychosis: An investigation of associations between general attachment styles and attachment relationships with specific others. Social Psychiatry and Psychiatric Epidemiology, 42, 972–976. 10.1007/s00127-007-0261-5 17932610

[papt12314-bib-0012] Bowlby, J. (1982). Attachment and loss: Vol. 1. Attachment (2nd. ed.). New York, NY: Basic Books.

[papt12314-bib-0013] Burns, C., & Conchie, S. (2015). Measuring implicit trust and automatic attitude. In Handbook of research methods on trust (pp. 239–248). Cheltenham: Edward Elgar Publishing.

[papt12314-bib-0014] Carr, S. C., Hardy, A., & Fornells‐Ambrojo, M. (2018). Relationship between attachment style and symptom severity across the psychosis spectrum: A meta‐analysis. Clinical Psychology Review, 59(November), 145–158. 10.1016/j.cpr.2017.12.001 29229220

[papt12314-bib-0015] Correll, J., Park, B., Judd, C. M., & Wittenbrink, B. (2002). The police officer’s dilemma: Using ethnicity to disambiguate potentially threatening individuals. Journal of Personality and Social Psychology, 83, 1314–1329. 10.1037//0022-3514.83.6.1314 12500813

[papt12314-bib-0016] Elahi, A., Perez Algorta, G., Varese, F., McIntyre, J. C., & Bentall, R. P. (2017). Do paranoid delusions exist on a continuum with subclinical paranoia? A multi‐method taxometric study. Schizophrenia Research, 190, 77–81. 10.1016/j.schres.2017.03.022 28318838

[papt12314-bib-0017] Essien, I., Stelter, M., Kalbe, F., Koehler, A., Mangels, J., & Meliß, S. (2017). The shooter bias: Replicating the classic effect and introducing a novel paradigm. Journal of Experimental Social Psychology, 70, 41–47. 10.1016/j.jesp.2016.12.009

[papt12314-bib-0018] Fett, A. K. J., Shergill, S. S., Korver‐Nieberg, N., Yakub, F., Gromann, P. M., & Krabbendam, L. (2016). Learning to trust: Trust and attachment in early psychosis. Psychological Medicine, 46, 1437–1447. 10.1017/S0033291716000015 26898947

[papt12314-bib-0019] Fox, J., & Weisberg, S. (2019). An (R) companion to applied regression. Thousand Oaks, CA: Sage. https://socialsciences.mcmaster.ca/jfox/Books/Companion/

[papt12314-bib-0020] Freeman, D. (2007). Suspicious minds: The psychology of persecutory delusions. Clinical Psychology Review, 27, 425–457. 10.1016/j.cpr.2006.10.004 17258852

[papt12314-bib-0021] Freeman, D., Garety, P. A., Bebbington, P. E., Smith, B., Rollinson, R., Fowler, D., … Dunn, G. (2005). Psychological investigation of the structure of paranoia. The British Journal of Psychiatry, 186(5), 427–435. 10.1192/bjp.186.5.427 15863749

[papt12314-bib-0022] Frith, C. D. (1979). Consciousness, information processing and schizophrenia. British Journal of Psychiatry, 134(3), 225–235. 10.1192/bjp.134.3.225 509004

[papt12314-bib-0023] Green, M. F., Penn, D. L., Bentall, R., Carpenter, W. T., Gaebel, W., Gur, R. C., … Heinssen, R. (2008). Social Cognition in schizophrenia: An NIMH workshop on definitions, assessment, and research opportunities. Schizophrenia Bulletin, 34, 1211–1220. 10.1093/schbul/sbm145 18184635 PMC2632490

[papt12314-bib-0024] Gumley, A. I., Taylor, H. E. F., Schwannauer, M., & MacBeth, A. (2014). A systematic review of attachment and psychosis: Measurement, construct validity and outcomes. Acta Psychiatrica Scandinavica, 129(4), 257–274. 10.1111/acps.12172 23834647

[papt12314-bib-0025] Hahn, A., & Gawronski, B. (2015). Implicit social cognition. International Encyclopedia of the Social & Behavioral Sciences, 4, 714–720. 10.1016/B978-0-08-097086-8.24066-X

[papt12314-bib-0026] Haselton, M. G., & Buss, D. M. (2000). Error management theory: A new perspective on biases in cross‐sex mind reading. Journal of Personality and Social Psychology, 78(1), 81–91. 10.1037/0022-3514.78.1.81 10653507

[papt12314-bib-0043] Haselton, M. G., & Nettle, D. (2006). The paranoid optimist: An integrative evolutionary model of cognitive biases. Personality and Social Psychology Review, 10(1), 47–66. 10.1207/s15327957pspr1001_3 16430328

[papt12314-bib-0027] Hlavac, M. (2018). Stargazer: Well‐formatted regression and summary statistics tables. R package version 5.2.1. Bratislava, Slovakia: Central European Labour Studies Institute (CELSI). https://CRAN.R‐project.org/package=stargazer

[papt12314-bib-0028] Hooker, C. I., Tully, L. M., Verosky, S. C., Fisher, M., Holland, C., & Vinogradov, S. (2011). Can I trust you? Negative affective priming influences social judgments in schizophrenia. Journal of Abnormal Psychology, 120(1), 98–107. 10.1037/a0020630 20919787 PMC3170843

[papt12314-bib-0029] Hu, L., & Bentler, P. M. (1999). Cutoff criteria for fit indexes in covariance structure analysis: Conventional criteria versus new alternatives. Structural Equation Modeling: A Multidisciplinary Journal, 6(1), 1–55. 10.1080/10705519909540118

[papt12314-bib-0030] Kenny, D. A. (2019). Measuring Model Fit. Retrieved from http://www.davidakenny.net/cm/fit.htm

[papt12314-bib-0031] King, G. (1986). How not to lie with statistics: Avoiding common mistakes in quantitative political science. American Journal of Political Science, 30, 666. 10.2307/2111095

[papt12314-bib-0032] Kirk, H., Gilmour, A., Dudley, R., & Riby, D. (2013). Paranoid ideation and assessments of trust. Journal of Experimental Psychopathology, 4, 360–367. 10.5127/jep.027812

[papt12314-bib-0033] Kline, R. B. (2015). Principles and practice of structural equation modeling. New York, NY: Guilford.

[papt12314-bib-0034] Lecomte, T., Corbière, M., & Laisné, F. (2006). Investigating self‐esteem in individuals with schizophrenia: Relevance of the Self‐Esteem Rating Scale‐Short Form. Psychiatry Research, 143(1), 99–108. 10.1016/j.psychres.2005.08.019 16725210

[papt12314-bib-0035] Lee, A., & Hankin, B. L. (2009). Insecure attachment, dysfunctional attitudes, and low self‐esteem predicting prospective symptoms of depression and anxiety during adolescence. Journal of Clinical Child and Adolescent Psychology, 38(2), 219–231. 10.1080/15374410802698396 19283600 PMC2741157

[papt12314-bib-0036] Lee, I. A., & Preacher, K. J. (2013). Calculation for the test of the difference between two dependent correlations with no variable in common [Computer software]. Retrieved from http://quantpsy.org/corrtest/corrtest3.htm

[papt12314-bib-0037] Lynn, S. K., & Barrett, L. F. (2014). “Utilizing” signal detection theory. Psychological Science, 25, 1663–1673. 10.1177/0956797614541991 25097061 PMC4304641

[papt12314-bib-0038] MacCallum, R. C., Browne, M. W., & Sugawara, H. M. (1996). Power analysis and determination of sample size for covariance structure modeling. Psychological Methods, 1(2), 130–149. 10.1037/1082-989X.1.2.130

[papt12314-bib-0039] Mekawi, Y., & Bresin, K. (2015). Is the evidence from racial bias shooting task studies a smoking gun? Results from a meta‐analysis. Journal of Experimental Social Psychology, 61(September), 120–130. 10.1016/j.jesp.2015.08.002

[papt12314-bib-0040] Melo, S., Corcoran, R., Shryane, N., & Bentall, R. P. (2009). The persecution and deservedness scale. Psychology and Psychotherapy: Theory, Research and Practice, 82(3), 247–260. 10.1348/147608308X398337 19426584

[papt12314-bib-0041] Mikulincer, M. (1995). Attachment style and the mental representation of the self. Journal of Personality and Social Psychology, 69, 1203–1215. 10.1037/0022-3514.69.6.1203

[papt12314-bib-0042] Mikulincer, M. (1998). Attachment working models and the sense of trust: An exploration of interaction goals and affect regulation. Journal of Personality and Social Psychology, 74(5), 209–224. 10.1037/0022-3514.74.5.1209

[papt12314-bib-0044] Nugent, W. R., & Thomas, J. W. (1993). Validation of a clinical measure of self‐esteem. Research on Social Work Practice, 3(2), 191–207. 10.1177/104973159300300205

[papt12314-bib-0045] Oosterhof, N. N., & Todorov, A. (2008). The functional basis of face evaluation. Proceedings of the National Academy of Sciences, 105, 11087–11092. 10.1073/pnas.0805664105 PMC2516255 18685089

[papt12314-bib-0046] Pickering, L., Simpson, J., & Bentall, R. P. (2008). Insecure attachment predicts proneness to paranoia but not hallucinations. Personality and Individual Differences, 44(5), 1212–1224. 10.1016/j.paid.2007.11.016

[papt12314-bib-0047] R Core Team (2018). R: A language and environment for statistical computing. Vienna, Austria: R Foundation for Statistical Computing. http://www.r‐project.org/

[papt12314-bib-0048] Ringer, J. M., Buchanan, E. E., Olesek, K., & Lysaker, P. H. (2014). Anxious and avoidant attachment styles and indicators of recovery in schizophrenia: Associations with self‐esteem and hope. Psychology and Psychotherapy: Theory, Research and Practice, 87(2), 209–221. 10.1111/papt.12012 23913519

[papt12314-bib-0049] Roberts, J. E., Gotlib, I. H., & Kassel, J. D. (1996). Adult attachment security and symptoms of depression: The mediating roles of dysfunctional attitudes and low self‐esteem. Journal of Personality and Social Psychology, 70, 310–320. 10.1016/S0272-6386(97)70025-8 8636884

[papt12314-bib-0050] RStudio Team (2016). RStudio: Integrated development environment for R. Boston, MA: RStudio Inc. http://www.rstudio.com/

[papt12314-bib-0051] Shaver, P. R., & Mikulincer, M. (2005). Attachment theory and research: Resurrection of the psychodynamic approach to personality. Journal of Research in Personality, 39(1), 22–45. 10.1016/j.jrp.2004.09.002

[papt12314-bib-0052] Sitko, K., Bentall, R. P., Shevlin, M., O’Sullivan, N., & Sellwood, W. (2014). Associations between specific psychotic symptoms and specific childhood adversities are mediated by attachment styles: An analysis of the National Comorbidity Survey. Psychiatry Research, 217(3), 202–209. 10.1016/j.psychres.2014.03.019 24726818

[papt12314-bib-0053] Sitko, K., Varese, F., Sellwood, W., Hammond, A., & Bentall, R. (2016). The dynamics of attachment insecurity and paranoid thoughts: An experience sampling study. Psychiatry Research, 246, 32–38. 10.1016/j.psychres.2016.08.057 27649527

[papt12314-bib-0054] Stanislaw, H., & Todorov, N. (1999). Calculation of signal detection theory measures. Behavior Research Methods, Instruments, & Computers, 3(I), 37–149. 10.3758/BF03207704 10495845

[papt12314-bib-0055] Sutcliffe, A., Dunbar, R., Binder, J., & Arrow, H. (2012). Relationships and the social brain: Integrating psychological and evolutionary perspectives. British Journal of Psychology, 103(2), 149–168. 10.1111/j.2044-8295.2011.02074.x 22506741

[papt12314-bib-0056] Swets, J. A., Dawes, R. M., & Monahan, J. (2000). Psychological science can improve diagnostic criteria. Psychological Science in the Public Interest, 1(1), 1–26. 10.1111/1529-1006.001 26151979

[papt12314-bib-0057] Thewissen, V., Bentall, R. P., Lecomte, T., van Os, J., & Myin‐Germeys, I. (2008). Fluctuations in self‐esteem and paranoia in the context of daily life. Journal of Abnormal Psychology, 117(1), 143–153. 10.1037/0021-843X.117.1.143 18266492

[papt12314-bib-0058] Todorov, A., Dotsch, R., Porter, J. M., Oosterhof, N. N., & Falvello, V. B. (2013). Validation of data‐driven computational models of social perception of faces. Emotion, 13, 724–738. 10.1037/a0032335 23627724

[papt12314-bib-0059] Todorov, A., Pakrashi, M., & Oosterhof, N. N. (2009). Evaluating faces on trustworthiness after minimal time exposure. Social Cognition, 27, 813–833. 10.1521/soco.2009.27.6.813

[papt12314-bib-0060] Tsoi, D. T., Lee, K. H., Khokhar, W. A., Mir, N. U., Swalli, J. S., Gee, K. A., … Woodruff, P. W. R. (2008). Is facial emotion recognition impairment in schizophrenia identical for different emotions? A signal detection analysis. Schizophrenia Research, 99(1–3), 263–269. 10.1016/j.schres.2007.11.006 18180142

[papt12314-bib-0061] Varese, F., Smeets, F., Drukker, M., Lieverse, R., Lataster, T., Viechtbauer, W., … Bentall, R. P. (2012). Childhood adversities increase the risk of psychosis: A meta‐analysis of patient‐control, prospective‐and cross‐sectional cohort studies. Schizophrenia Bulletin, 38, 661–671. 10.1093/schbul/sbs050 22461484 PMC3406538

[papt12314-bib-0062] Westermann, S., & Lincoln, T. M. (2010). Using signal detection theory to test the impact of negative emotion on sub‐clinical paranoia. Journal of Behavior Therapy and Experimental Psychiatry, 41(2), 96–101. 10.1016/j.jbtep.2009.10.007 19931042

[papt12314-bib-0063] Wickham, S., Sitko, K., & Bentall, R. P. (2015). Insecure attachment is associated with paranoia but not hallucinations in psychotic patients: The mediating role of negative self‐esteem. Psychological Medicine, 45, 1495–1507. 10.1017/S0033291714002633 25388512

