# Supplementary material for: Mistrust and negative self‐esteem: Two paths from attachment styles to paranoia
Source: Psychol Psychother. 2020 Dec 13;94(3):391–406. doi: 10.1111/papt.12314 (PMC8451824; doi:10.1111/papt.12314)
Supplement: Supplementary file 1 — Figure S1. The only significant indirect effect was attachment anxiety → negative self‐esteem → paranoia, β = .065, 95% CI 0.055–0.077, p < .001. [file PAPT-94-391-s001.docx]

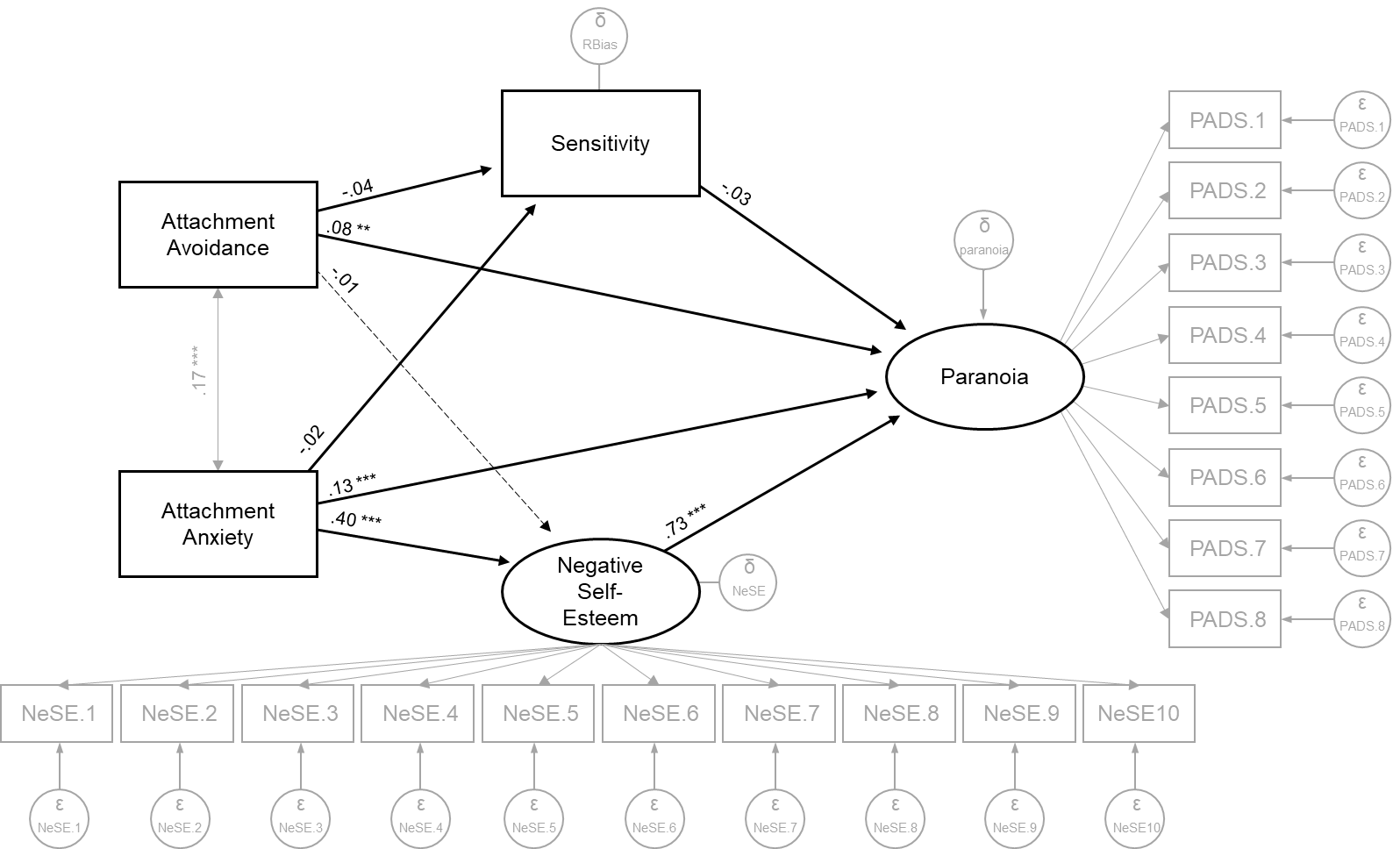


*Figure S1*. The only significant indirect effect was attachment anxiety 🡪 negative self-esteem 🡪 paranoia, β = 0.065, 95% CI
0.055 - 0.077, p <.001. None of the effects went through the sensitivity measure.
